# Saturated Red Electroluminescence From Thermally Activated Delayed Fluorescence Conjugated Polymers

**DOI:** 10.3389/fchem.2020.00332

**Published:** 2020-04-24

**Authors:** Hongmei Zhan, Yanjie Wang, Kuofei Li, Yuannan Chen, Xiaohu Yi, Keyan Bai, Guohua Xie, Yanxiang Cheng

**Affiliations:** ^1^State Key Laboratory of Polymer Physics and Chemistry, Changchun Institute of Applied Chemistry, Chinese Academy of Sciences, Changchun, China; ^2^Sauvage Center for Molecular Sciences, Hubei Key Lab on Organic and Polymeric Optoelectronic Materials, Department of Chemistry, Wuhan University, Wuhan, China; ^3^Guangdong Provincial Key Laboratory of Luminescence From Molecular Aggregates (South China University of Technology), Guangzhou, China

**Keywords:** thermally activated delayed fluorescence, red emission, conjugated polymers, anthraquinone, electroluminescence

## Abstract

Two sets of conjugated polymers with anthraquinone groups as pendant acceptors were designed and synthesized. The acceptor is tethered to an diphenylamine group via a phenylene bridge, constructing a thermally activated delayed fluorescence (TADF) unit, which is embedded into the polymer backbone through its donor fragment, while the backbone is composed of dibenzothiophene-*S, S*-dioxide and 2, 7-fluorene or 2, 7-carbazole groups. The polymers show distinct TADF characteristics, confirmed by transient photoluminescence spectra and theoretical calculations. The carbazole-based polymers exhibit shorter delay lifetimes and lower energy emission relative to the fluorene-based polymers. The non-doped organic light-emitting diodes fabricated via solution processing approach produce efficient red emissions with the wavelengths of 625–646 nm. The carbazole containing polymer with 2% molar content of the TADF unit exhibits the best maximum external quantum efficiency of 13.6% and saturated red electroluminescence with the Commission Internationale de l'Eclairage coordinates of (0.62, 0.37).

## Introduction

As the new-generation luminescent materials, metal-free thermally activated delayed fluorescence (TADF) emitters have drawn great attentions in the field of organic light-emitting diodes (OLEDs) because they could theoretically achieve 100% internal quantum efficiency through reverse intersystem crossing (RISC) process of non-radiative triplet excitons (Tao et al., 2014; Im et al., 2017; Yang et al., 2017; Huang et al., 2018; Liu et al., 2018; Zou et al., 2018; Godumala et al., 2019). Since Uoyama et al. accomplished a great breakthrough in TADF OLEDs in 2012, realizing the maximum external quantum efficiency (EQE) close to 20% (Uoyama et al., 2012), significant progresses in the development of novel materials and device engineering have been committed, especially in blue and green TADF OLEDs with the EQEs even over 30% (Lin et al., 2016; Wu et al., 2018). However, as one of the three primary colors, the electroluminescent (EL) performances of red TADF emitters still lag far behind due to high non-radiative transition rates and serious concentration quenching effect (Kim et al., 2018a,b; Chen et al., 2019; Wang et al., 2019; Zeng et al., 2019). In addition, the currently reported red TADF materials are mainly based on organic small molecules with the twisted donor/acceptor structures (Furue et al., 2018; Zhang et al., 2019). In contrast, red TADF polymer has rarely been reported so far, partially due to the challenging material design and synthesis (Wang et al., 2018; Yang et al., 2018a). In view of the good solubility and film-forming property, polymers are more suitable for solution process, which is advantageous for the preparation of low-cost, large area, and non-doped devices (Xie and Li, 2017; Zhang and Cheng, 2019). Therefore, it is urgent to develop efficient red TADF polymers with some new molecular structures.

Conjugated polymer can form a large conjugated system due to the π-electron delocalization in backbone. Theoretically, it is easier to obtain a small energy gap and thus realize red emission. However, the traditional conjugated polymers with conjugation along backbone generally lead to fluorescence rather than TADF due to the lack of the sufficiently twisted donor/acceptor structure. Therefore, a few novel polymeric structures have been presented to produce TADF effect. For example, a red TADF unit is attached on the poly(aryl ether) backbone through an alkyl chain and the resultant polymers exhibit red emission peaked at 606 nm with an EQE of up to 5.6% (Yang et al., 2018a). In our previous work, based on the backbone-donor/pendant-acceptor (BDPA) strategy (Zhu et al., 2016, 2018; Yang et al., 2018b, 2019), a series of long-wavelength emissive TADF conjugated polymers containing the narrow bandgap TADF unit 2-(4-(diphenylamino)-phenyl)-9*H*-thioxanthen-9-one-10,10-dioxide were also prepared (Wang Y. et al., 2017). The polymers not only inherited the inherent TADF characteristics of the small molecules as monomers, but also exhibited the red-shifted emission compared with the monomers. Among these polymers, PFSOTT2 showed bright orange emission and a considerably high maximum EQE close to 20%. These results motivate us to further develop efficient red TADF polymers by improving the polymer architecture design.

**Scheme 1:**
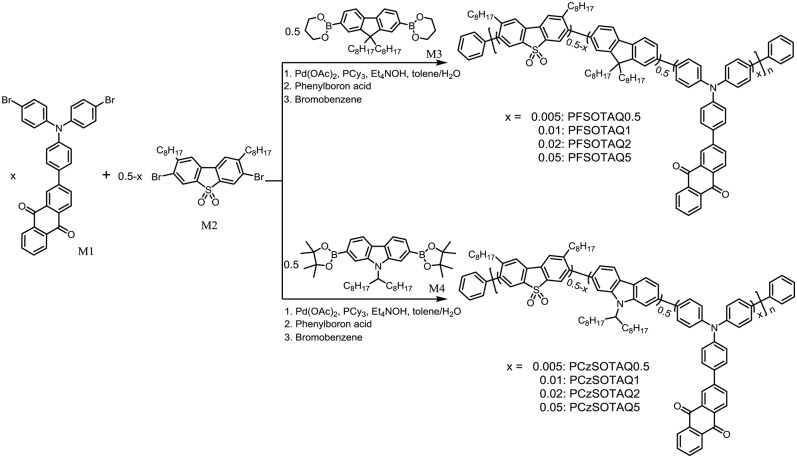
Chemical structures and synthetic routes of PFSOTAQx and PCzSOTAQx.

Herein, two series of TADF conjugated polymers PFSOTAQx and PCzSOTAQx (*x* = 0.5, 1, 2 and 5, respectively) were designed and synthesized based on the BDPA strategy (Scheme 1). A rigid anthraquinone group with the stronger electron-withdrawing ability, compared with 9*H*-thioxanthen-9-one-10,10-dioxide, is attached to the diphenylamine fragment of polymer backbone via a phenylene bridge to form a narrow bandgap TADF unit (TAQ, seemingly consisting of a triphenylamine donor and an anthraquinone acceptor). The apparent difference between two sets of the polymers originates from the polymer backbones, consisting of dibenzothiophene-*S, S*-dioxide (SO) and 2, 7-fluorene (F) or SO and 2, 7-carbazole (Cz) groups, respectively, in which the introduction of SO group can weaken the conjugation of the polymer backbone and thus raise the triplet energy level of the polymers (Wang Y. et al., 2017). In comparison with fluorene group, Cz ring has a stronger electron donating ability and acts a better hole transporting role. As expected, the carbazole-based polymers PCzSOTAQx display better device performances. Especially, PCzSOTAQ2 achieves the saturated red emission with the Commission Internationale de l'Eclairage (CIE) coordinates of (0.62, 0.37) and a maximum EQE of 13.6%.

## Results and Discussion

### Synthesis and Characterization

As shown in Scheme 1, polymers PFSOTAQx and PCzSOTAQx were synthesized via Suzuki polycondensation of the corresponding monomers, 2-(4-(bis(4-bromophenyl)amino)phenyl)-anthraquinone (M1), 3,7-dibromo-2,8-dioctyldibenzothiophene-S,S-dioxide (M2) and alkyl substituted fluorene or carbazole diboronic ester (M3 or M4), using Pd(OAc)_2_/PCy_3_ as catalyst and tetraethylammonium hydroxide (Et_4_NOH) as an emulsifying base, followed by end-capping with phenyl boronic acid and bromobenzene (Liu et al., 2008a). Higher molecular weight can be obtained under the reaction condition instead of Pd(P(*o*-tol)_3_)_2_Cl_2_/K_3_PO_4_/THF catalytic system generally used in the previous works (Zhu et al., 2016, 2018; Yang et al., 2018b, 2019). The molar feed ratio (x) of M1 was varied from 0.5 to 5%, and the corresponding polymers were named as PFSOTAQx and PCzSOTAQx, respectively. TAQ molecule was prepared by Suzuki coupling reaction of 2-bromoanthraquinone and 4-(diphenylamino)- phenylboronic acid, followed by bromination with *N*-bromosucccinimide (NBS) in the dark to afford the monomer M1 (Scheme S1). Monomers M2-M4 with alkyl substituents were synthesized according to the literatures (Xin et al., 2005; Blouin et al., 2007; Kamtekar et al., 2010). The introduction of alkyl groups allows the polymers to have excellent solubility in common organic solvents, such as THF, chloroform and toluene, etc., which is favorable for solution processing of light-emitting device. The number average molecular weights (*M*_n_) of PFSOTAQx range from 54.2 to 65.8 kDa with polydispersity indexes (PDIs) of 2.0–2.3, while PCzSOTAQx have the relatively low *M*_n_s of 25.4–39.1 kDa and similar PDI values (Table S1), as determined by gel permeation chromatography (GPC) with 1,2,4-trichlorobenzene as eluent and calibrated against polystyrene standards. All polymers show excellent thermal stability with a high decomposition temperature of over 426°C (T_d_, corresponding to 5% weight loss, Figure S1). The T_d_ values of PCzSOTAQx are higher than those of PFSOTAQx because carbazole is fully aromatic in contrast to fluorene group, providing a better chemical and environmental stability (Blouin et al., 2007). The film forming abilities of PFSOTAQ2 and PCzSOTAQ2 were also studied by atomic force microscopy (AFM) technology (Supporting Information). The root mean square (RMS) roughness of thin films is 0.193 nm for PFSOTAQ2 and 0.179 nm for PCzSOTAQ2, indicating that both film surfaces are quite smooth. The superior film morphologies further confirm that these polymers are suitable for preparing OLED devices via solution processing method.

### Photophysical Properties

**Figure 1:**
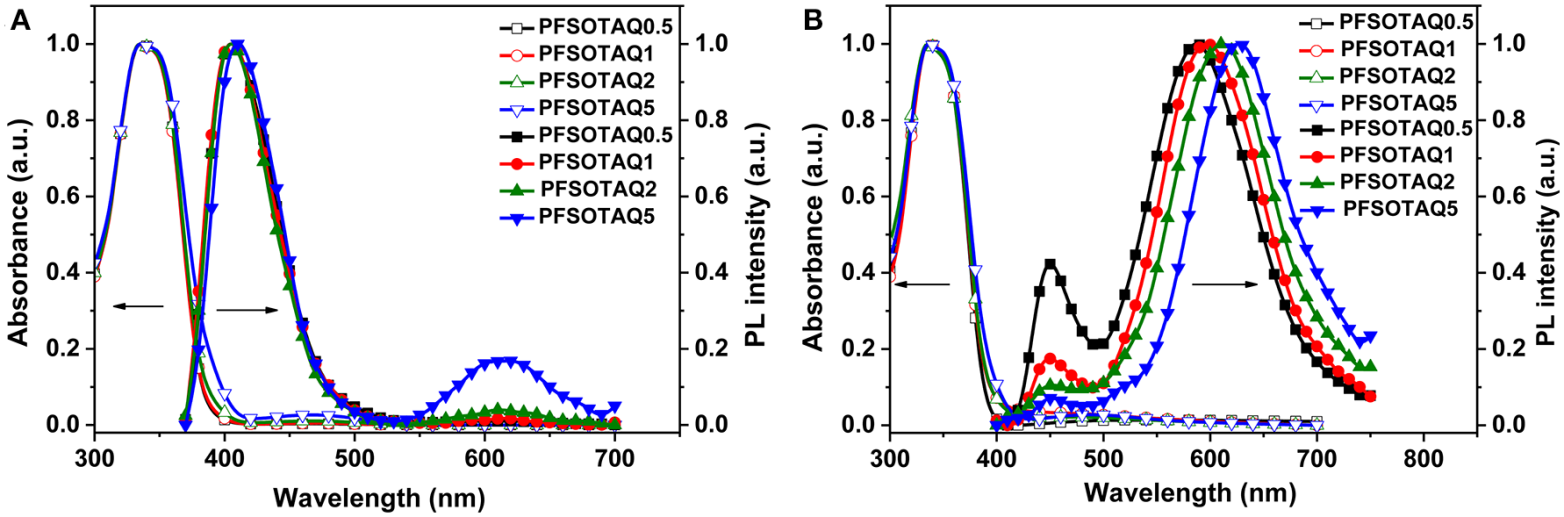
Normalized UV-vis absorption and PL spectra of PFSOTAQx in oxygen-free toluene **(A)** and neat film **(B)** at 298 K.

**Figure 2:**
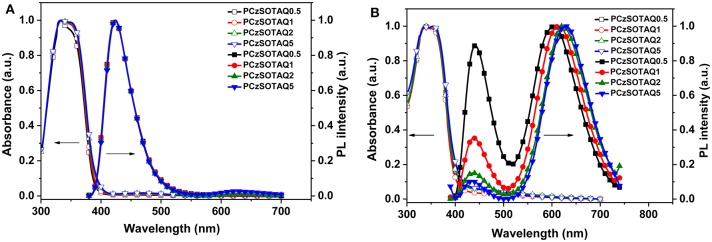
Normalized UV-vis absorption and PL spectra of PCzSOTAQx in oxygen-free toluene **(A)** and neat film **(B)** at 298 K.

The polymers PFSOTAQx and PCzSOTAQx exhibit similar UV-vis absorption and photoluminescence (PL) profiles either in dilute solution or neat films (Figures 1, 2). Taking PFSOTAQx as examples, the strong absorption bands at around 336 nm are ascribed to the π-π^*^ transition of the conjugated backbone, and the weak absorption band between 400 and 550 nm observed in PFSOTAQ2 and PFSOTAQ5 with the higher contents of the TAQ unit can be assigned to the intramolecular charge-transfer (ICT) transition. In dilute solution, PFSOTAQ1-PFSOTAQ5 display dual emissions with a dominant blue emission which peaks at around 405 nm and originates from the polymer backbone. Moreover, a weak red emission peaked at around 611 nm results from the TADF unit. In contrast, PFSOTAQ0.5 with a low content of the TAQ unit only exhibits a blue emission. Unlike their absorption spectra, the clearly intensified red emission, especially in PFSOTAQ5, indicates the occurrence of the intramolecular Förster energy transfer from the polymer backbone to the TADF unit. This is supported by the sufficient overlap between the PL emission of the backbone and the ICT absorption of the TADF unit (Figure S2). In neat film, the polymers exhibit a dominant red emission and a weak blue emission, indicating that the more efficient energy transfer from the backbone to the TADF unit occurs due to the synergistic effect of intramolecular and intermolecular interactions in the solid state. Concurrently, the red emission of PFSOTAQx gradually shifts from 587 to 627 nm as the content of the TADF unit increases owing to the enhanced aggregation of the TADF unit. In comparison with PFSOTAQx, the maximum emission peaks of PCzSOTAQx show some red shifts of 1-19 nm due to the stronger electron-donating ability of carbazole compared with the fluorene group. These results demonstrate that the red emission of conjugated polymer could be achieved simply by enhancing the strength of pendant acceptor and/or backbone donor.

**Figure 3:**
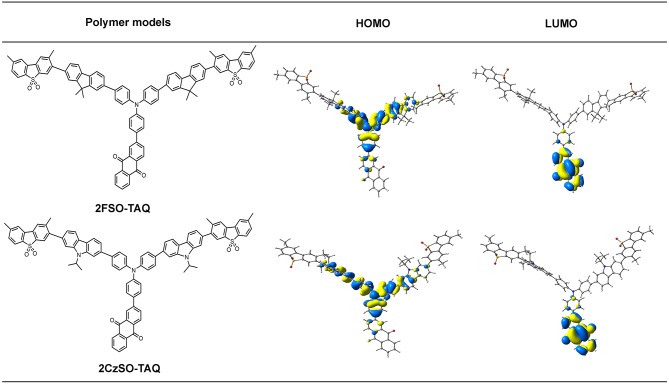
The molecular structures and HOMO/LUMO distributions of the polymer models in the S_0_ state. They were optimized at the DFT/BMK/6-31G(d) level.

In order to understand the relationship between the geometric structures and intrinsic electronic characteristics of the polymers, time-dependent density functional theory (TD-DFT) calculations were performed. 2FSO-TAQ and 2CzSO-TAQ containing one TADF unit, two fluorene or carbazole and two dibenzothiophene-S,S-dioxide rings were chosen as the polymer models. Their ground-state (S_0_) geometries were optimized at the BMK/6-31G(d) level in toluene. In the S_0_ geometries, the dihedral angles between the donor and phenylene bridge are 36.5° for 2FSO-TAQ and 38.0° for 2CzSO-TAQ (Figure S7), which are similar to those of TADF small molecules based on the diphenylamine donor (Zhang et al., 2014; Bin et al., 2017; Wang S. et al., 2017; Furue et al., 2018), indicating that the change of the backbone building segment has no significant effect on the twisted angle between the acceptor and donor of the TADF unit, and that the polymers could inherit the TADF characteristics of the TADF unit. As shown in Figure 3, the lowest unoccupied molecular orbitals (LUMOs) of 2FSO-TAQ and 2CzSO-TAQ are predominantly located on the anthraquinone acceptor, whereas the highest occupied molecular orbitals (HOMOs) are mainly distributed on the diphenylamine donor, the adjacent fluorene or carbazole rings and phenylene bridge. There is a certain degree of overlap on the phenylene bridge. The calculated singlet-triplet energy gaps (Δ*E*_ST_s) are 0.20 eV for 2FSO-TAQ and 0.16 eV for 2CzSO-TAQ, which are good agreement with the experimental values of PFSOTAQ2 and PCzSOTAQ2 in film (Figure S3 and Tables S2, S3). The small Δ*E*_ST_ values are in favor of the up-conversion from T_1_ to S_1_ states. In comparison with PFSOTAQx, the polymers PCzSOTAQx show the smaller Δ*E*_ST_, attributed to the stronger electron-donating ability of carbazole group. Additionally, 2CzSO-TAQ shows a slightly large oscillator strength (*f* ) relative to 2FSO-TAQ, which is beneficial to increase the fluorescence radiative rate (Zhang et al., 2014; Furue et al., 2018). This is also reflected that in the neat film the Φ_PL_s are up to 0.71 for PFSOTAQx and 0.75 for PCzSOTAQx, respectively.

**Figure 4:**
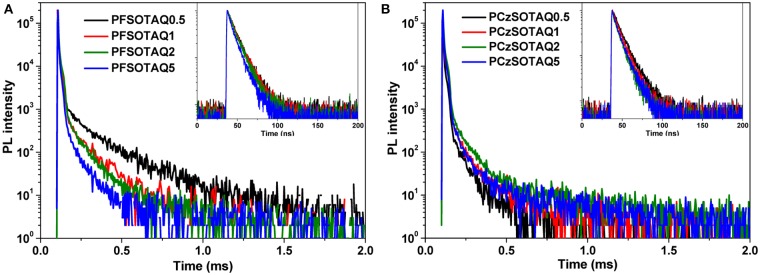
Transient PL decay spectra of PFSOTAQx **(A)** and PCzSOTAQx **(B)** in neat film at 298 K. Inset: Transient PL decay spectra measured in a time range of 200 ns.

To verify the TADF features of the polymers, the transient PL decay spectra were measured in neat film under nitrogen. As depicted in Figure 4 and Table 1, both PFSOTAQx and PCzSOTAQx show distinct nanosecond-scale prompt decay and microsecond-scale delayed decay. The lifetimes of the delayed fluorescence components (τ_DF_) of PCzSOTAQx significantly decrease, compared with those of PFSOTAQx, probably benefited from their smaller Δ*E*_ST_s. Meanwhile, PCzSOTAQx exhibits an increased prompt fluorescence rate constant (*k*_F_) and a higher delayed fluorescence rate (*k*_TADF_) relative to PFSOTAQx under the same molar content of the TAQ unit (Table S5, Supporting Information), which are in favor of effective utilization of triplet excitons and good device performance.

**Table 1:** Photophysical data for polymers PFSOTAQx and PCzSOTAQx.

**Polymers**	**λ_abs_**	**λ_PL_**	**Φ_PL_**	**τ_PF_ (ratio)**	**τ_DF_ (ratio)**	**HOMO**	**LUMO**
	**[nm]sol^**a**^/film^**b**^**	**[nm]sol^**a**^/film^**b**^**	**[%]^**c**^**	**[ns (%)]^**d**^**	**[μs (%)]^**d**^**	**[eV]^**e**^**	**[eV]^**f**^**
PFSOTAQ0.5	335/337	405/451, 587	70	10.8 (82)	226 (18)	−6.19	–
PFSOTAQ1	336/336	405,611/450,597	71	10.9 (84)	125 (16)	−6.17	–
PFSOTAQ2	336/336	405,611/443,612	58	10.2 (85)	93 (15)	−6.16	−4.12
PFSOTAQ5	337/337	410,615/451,627	39	8.9 (92)	83 (8)	−6.13	−4.09
PCzSOTAQ0.5	332/336	423/442,606	75	10.8 (94)	57 (6)	−5.92	–
PCzSOTAQ1	335/336	424,632/439,615	62	9.8 (89)	85 (11)	−5.91	–
PCzSOTAQ2	335/337	424,627/437,623	53	8.9 (86)	89 (14)	−5.90	−3.86
PCzSOTAQ5	335/355	424,625/434,628	48	8.8 (90)	60 (10)	−5.89	−3.85

a *Measured in toluene at room temperature*.

b *Measured in neat film at room temperature*.

c *Measured in the neat film free of oxygen*.

d *The lifetimes and fractional contributions of the prompt (τ_PF_) and delayed (τ_DF_) decay components in neat film measured at 298 K*.

e *Determined by cyclic voltammetry in neat films*.

f *Deduced from the HOMO and the optical energy gap (E_g_) values*.

### Electroluminescent Properties

**Figure 5:**
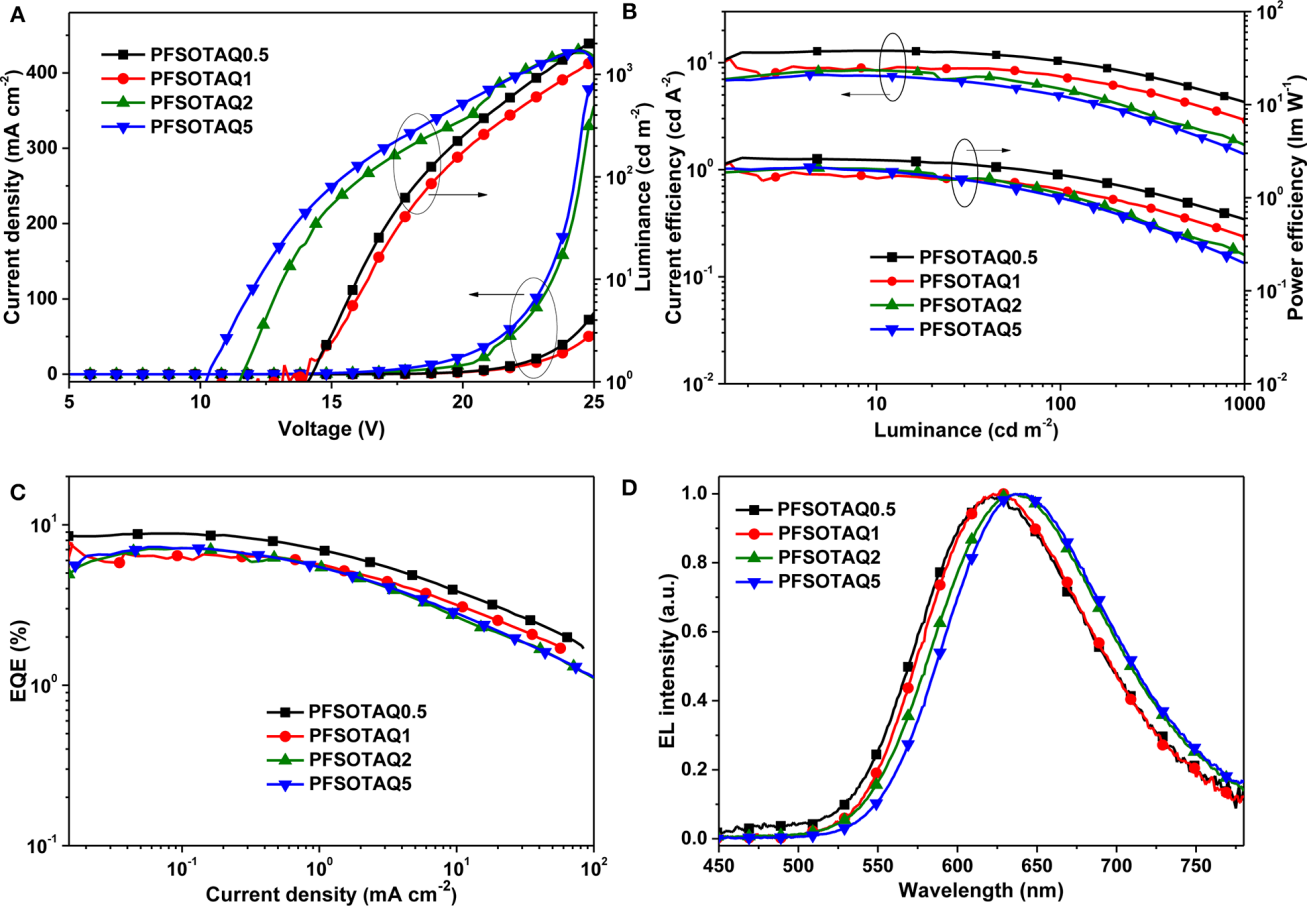
EL performance of non-doped OLED devices based on PFSOTAQx: **(A)** current density-voltage-luminance (*J*-*V*-*L*), **(B)** current efficiency-luminance-power efficiency (CE-L-PE) characteristics, **(C)** EQE-current density plots, and **(D)** EL spectra at the bias of 12V.

**Figure 6:**
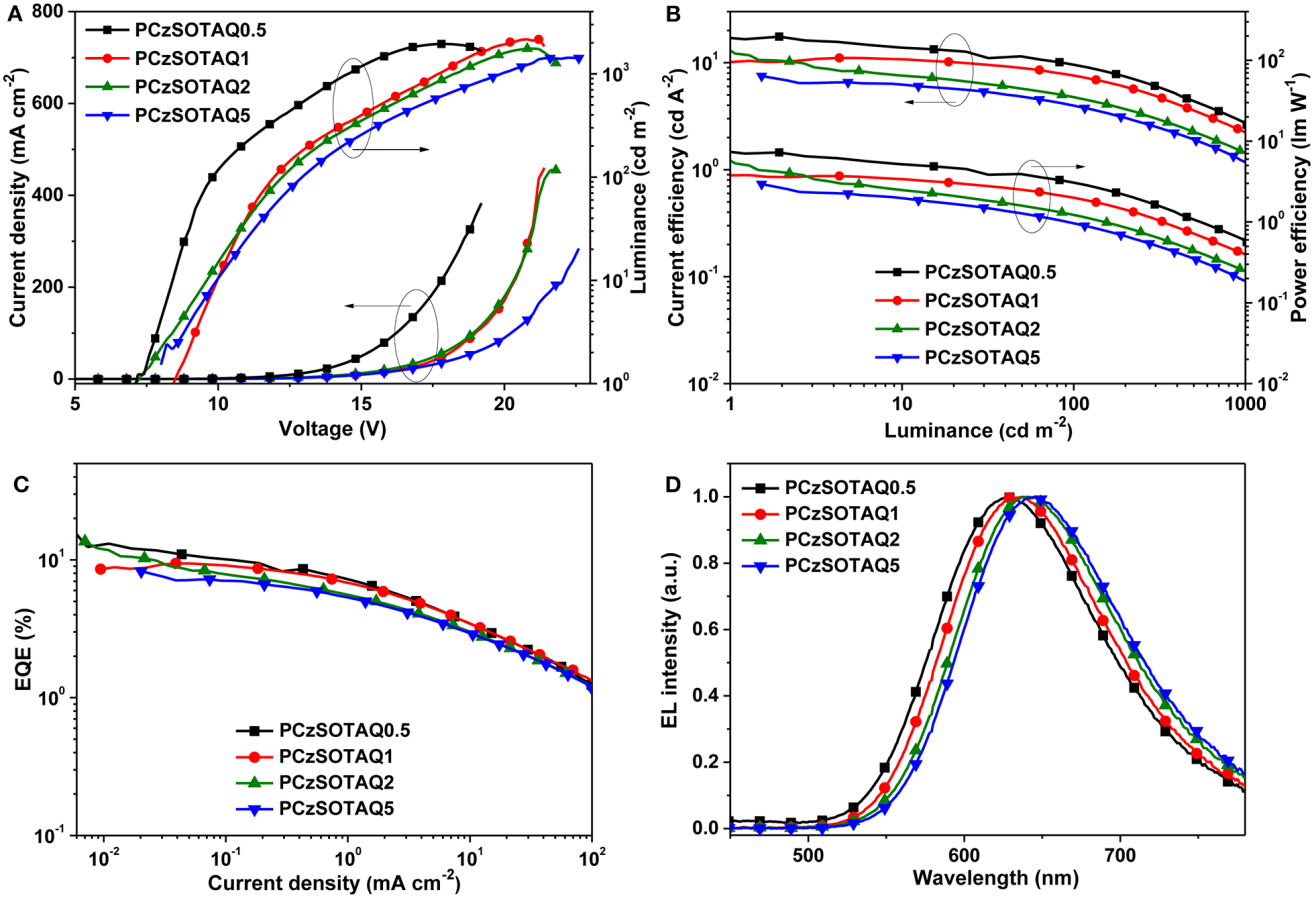
EL performance of non-doped OLED devices based on PCzSOTAQx: **(A)** current density-voltage-luminance (*J*-*V*-*L*), **(B)** current efficiency-luminance-power efficiency (CE-L-PE) characteristics, **(C)** EQE-current density plots, and **(D)** EL spectra at the bias of 12V.

To evaluate their EL, the non-doped OLEDs were fabricated with a structure of ITO/PEDOT:PSS (40 nm)/PFSOTAQx or PCzSOTAQx (40 nm)/SPPO13 (70 nm)/Liq (1 nm)/Al (100 nm), where PEDOT:PSS [poly(3,4-ethylenedioxythiophene):poly(styrene sulfonate)], Liq (8-hydroxyquinolinolato-lithium), and SPPO13 (2,7-bis(diphenylphosphoryl)-9,9′ -spirobifluorene) were used as the hole-injection layer, the electron-injection layer and the electron-transporting layer, respectively. Figures 5, 6 show the characteristic EL curves, and the key devices parameters are summarized in Table 2. All devices exhibit predominant red EL emission with the single peak at 625–638 nm for PFSOTAQx and 630–646 nm for PCzSOTAQx, in which the emissive peaks gradually red-shift with the increasing content of the TAQ unit, which is consistent with dominant emissive peaks of these polymers in PL spectra of neat film. There is no residual blue EL emission from the polymer backbones, even at 0.5 mol% TAQ content, which totally differs from the corresponding PL spectra. Such dramatic difference between the EL and PL spectra may be partially attributed to the direct charge trapping of the TADF unit in terms of the fact that the HOMO and LUMO energy levels of the model compound TAQ lie between those of PFSO or PCzSO (Figure S6; Gong et al., 2003), in addition to more effective energy transfer from the backbone to TAQ unit in the solid state. For PCzSOTAQx, the significant changes in blue emission peaks from the PL to EL spectra indicate that direct charge trapping on the TADF unit serves as the dominant EL mechanism rather than Förster energy transfer (Liu et al., 2008b; Ma et al., 2010). It can be further confirmed by the current density-voltage-luminance curves of the polymers. Moreover, in the same set of polymers, the variation trends of EL efficiencies basically correspond to the proportions of delayed fluorescence component, demonstrating that the EL process in the polymers comes from the TADF mechanism.

**Table 2:** EL data of the non-doped devices based on PFSOTAQx and PCzSOTAQx.

**Devices**	**$V_{\text{on}}^{\text{a}}$** **(V)**	**$L_{\text{max}}^{\text{b}}$** **(cd m^**−2**^)**	**${\mathrm{CE}}_{\text{max}}^{\text{c}}$** **(cd A^**−1**^)**	**${\mathrm{EQE}}_{\text{max}}^{\text{d}}$** **(%)**	**${\mathrm{PE}}_{\text{max}}^{\text{e}}$** **(lm W^**−1**^)**	**EL^**f**^** **(nm)**	**CIE** **(x, y)**
PFSOTAQ0.5	14.1	2,107	12.9	8.8	2.7	625	(0.57, 0.41)
PFSOTAQ1	13.8	1,365	10.8	7.8	2.4	627	(0.59, 0.40)
PFSOTAQ2	11.5	1,716	8.5	7.2	2.1	637	(0.60, 0.39)
PFSOTAQ5	10.2	1,708	7.7	7.3	2.1	638	(0.62, 0.38)
PCzSOTAQ0.5	7.1	1,947	17.5	13.1	7.2	630	(0.59, 0.39)
PCzSOTAQ1	8.4	2,158	11.2	9.5	3.7	637	(0.61, 0.39)
PCzSOTAQ2	7.1	1,753	12.2	13.6	5.3	642	(0.62, 0.37)
PCzSOTAQ5	7.7	1,434	7.5	8.3	2.9	646	(0.63, 0.37)

^a^*The turn-on voltage (V_on_)*.

^b^*The maximum luminance brightness (L_max_)*.

^c^*The maximum current efficiency (CE_max_)*.

^d^*The maximum external quantum efficiency (EQE_max_)*.

^e^*The maximum power efficiency (PE_max_)*.

f *The peak wavelength of EL spectra*.

By replacing fluorene in the polymer backbone with a 2,7-carbazole group, the polymers PCzSOTAQx exhibit superior EL performances compared with PFSOTAQx, complemented by the excellent hole transporting ability of carbazole group. The turn-on voltages of the devices are significantly reduced from 10.2–14.1 V to 7.1–8.5 V because the shallow HOMO energy levels of PCzSOTAQx reduce the injection barrier of holes from PEDOT:PSS. The maximum EQEs of all devices are over 7% while the CIE coordinates for PCzSOTAQ1-5 are (0.61, 0.39), (0.62, 0.37), and (0.63, 0.37), respectively.

Among these polymers, PCzSOTAQ2 achieves the highest maximum EQE of 13.6%. To the best of our knowledge, it is the best one of the solution-processed OLEDs based on red TADF polymers reported so far (Wang et al., 2018; Yang et al., 2018a; Hu et al., 2019). It is worth noting that our designed conjugated polymers show much better EL performances than the previously reported polymers PFDMPE-R01-10 with the TAQ unit [EQE: 5.6% and CIE: (0.57, 0.42)] (Yang et al., 2018a). To confirm the effect of carbazole group on the EL behavior, the hole and electron transporting abilities of PFSOTAQ2 and PCzSOTAQ2 were also investigated by the preparation of single carrier devices, as shown in Figure S7. Clearly, the carbazole-based PCzSOTAQ2 exhibits better hole transport capability, ascribed to the shallower HOMO energy level of PCzSOTAQ2, relative to the fluorene-based polymer PFSOTAQ2, while they show similar electron transport behaviors due to the same acceptor group and the similar conjugation. This may be one of the reasons for better comprehensive device performance of PCzSOTAQ2 than PFSOTAQ2. These results indicate that the backbone structure has a crucial influence on the EL behaviors of the polymers, and thus confirm that the conjugated structure is more favorable for obtaining more efficient red TADF polymer.

## Conclusions

In summary, the strong electron-withdrawing group anthraquinone was employed as pendant acceptor to successfully prepare red TADF conjugated polymers. Although the polymers all exhibited obvious the delayed fluorescence with microsecond-scale lifetimes, the introduction of carbazole group, instead of fluorene of the polymer backbone, created a positive effect on the PL and EL behaviors. The highest PL quantum yield in neat film is up to 0.75 and the maximum EQEs in the non-doped solution-processed devices all exceed the upper limit of 5% of the traditional fluorescent OLEDs. Especially, the device using the carbazole-based polymer PCzSOTAQ2 displays the saturated red EL emission with the CIE coordinates of (0.62, 0.37) and a very promising EQE of 13.6%. These results demonstrated that conjugated polymers have great potential as red TADF materials in the field of OLEDs and their PL and EL properties can be effectively improved through simple modification of the polymer structure.

## Data Availability Statement

All datasets generated for this study are included in the article/Supplementary Material.

## Author Contributions

HZ, YW, and KL performed the synthesis and characterization of materials, and photophysical properties measurements. YuC performed the theoretical calculation. KB carried out the EL experiments. HZ prepared the manuscript. HZ, XY, GX, and YaC conducted the discussion and analysis of the experimental results as well as the improvement of the manuscript.

## Conflict of Interest

The authors declare that the research was conducted in the absence of any commercial or financial relationships that could be construed as a potential conflict of interest.
