# Supplementary material for: Saturated Red Electroluminescence From Thermally Activated Delayed Fluorescence Conjugated Polymers
Source: Front Chem. 2020 Apr 24;8:332. doi: 10.3389/fchem.2020.00332 (PMC7212419; doi:10.3389/fchem.2020.00332)
Supplement: Supplementary file 1 [file Table_1.doc]

***Supporting Material***

**EXPERIMENTAL SECTION**

**Methods and Materials**

All chemicals and reagents were used as received from commercial sources without further purification. Anhydrous solvents (toluene and THF) were prepared by sodium treatment and distillation. 3,7-Dibromo-2,8-dioctyldibenzothiophene-S,S-dioxide (M2),[1] 9,9-dioctylfluorene- 2,7-bis(trimethylene boronates) (M3)[2] were prepared according to procedures described in the literature. ^1^H NMR and ^13^C NMR spectra were recorded with Bruker Avance 400 NMR spectrometers. MALDI-TOF mass spectra were performed on an AXIMA CFR MS apparatus (COMPACT). Molecular weight of the polymers were determined by gel permeation chromatography (GPC) on a Waters 410 instrument with polystyrene as a standard and 1,2,4-trichlorobenzene as the eluent. Thermogravimetric analysis (TGA) was performed with a Perkin-Elmer TGA-7 instrument. The thermal stability of the samples was determined under a nitrogen atmosphere at a heating rate of 10 °C min^−1^ from 25 to 800 °C.

**Materials Synthesis**


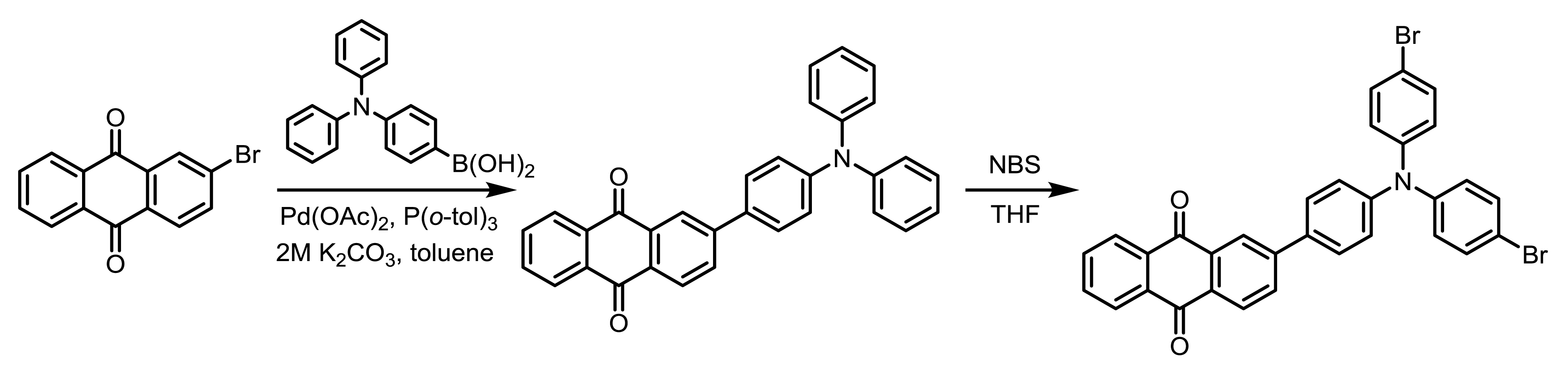


**Scheme S1**. Synthetic pathway of the TAQ monomer.

**2-(4-(Diphenylamino)phenyl)anthraquinone**

2-Bromoanthraquinone (1.4 g, 5 mmol), 4-(diphenylamino)phenylboronic acid (1.7 g, 6 mmol), palladium acetate Pd(OAc)_2_ (45 mg, 0.2 mmol), tri(*o*-tolyl)phosphine (121.7 mg, 0.4 mmol), 2M K_2_CO_3_ aqueous solution (5 mL) and toluene (10 mL) were added into a flask and purged with argon. The mixture was stirred at 80 °C overnight. After cooling, the product was extracted with CH_2_Cl_2_, washed with water and brine, and dried over anhydrous sodium sulfate. The organic layer was evaporated and the compound was purified by flash chromatography on silica gel column (petroleum ether: CH_2_Cl_2_ = 2:1) to give a red solid (1.8 g, 78%). ^1^H NMR (400 MHz, CDCl_3_) δ 8.51 (d, *J* = 1.7 Hz, 1H), 8.37–8.31 (m, 3H), 7.99 (dd, *J* = 8.2, 1.8 Hz, 1H), 7.84–7.78 (m, 2H), 7.62 (d, *J* = 8.6 Hz, 2H), 7.30 (t, *J* = 7.8 Hz, 4H), 7.16 (d, *J* = 6.6 Hz, 6H), 7.09 (t, *J* = 7.3 Hz, 2H). ^13^C NMR (101 MHz, CDCl_3_) δ 183.33, 182.79, 148.83, 147.28, 146.31, 134.09, 133.96, 133.91, 133.79, 133.70, 131.93, 131.59, 131.47, 129.44, 128.07, 128.02, 127.23, 127.17, 125.04, 124.71, 123.64, 122.94.

**2-(4-(Bis(4-bromophenyl)amino)phenyl)anthraquinone**

2-(4-(Diphenylamino)phenyl)anthraquinone (1.0 g, 2 mmol) was dissolved in anhydrous THF (30 mL), NBS (0.7 g, 4 mmol) was then added to the solution with small portions and the mixture was stirred overnight at room temperature in the dark. Until the reaction completion as shown by TLC analyses, the solvent was removed. The residue was purified by column chromatography on silica gel eluting with petroleum ether/CH_2_Cl_2_ (2:1) and re-crystallized from CH_2_Cl_2_/ethanol to give orange cotton-like solid (1.2 g, 96%). ^1^H NMR (400 MHz, CDCl_3_) δ 8.51 (s, 1H), 8.35 (dd, *J* = 11.7, 6.9 Hz, 3H), 7.98 (dd, *J* = 8.1, 1.5 Hz, 1H), 7.85–7.78 (m, 2H), 7.64 (d, *J* = 8.5 Hz, 2H), 7.40 (d, *J* = 8.7 Hz, 4H), 7.16 (d, *J* = 8.5 Hz, 2H), 7.01 (d, *J* = 8.7 Hz, 4H). ^13^C NMR (101 MHz, CDCl_3_) δ 183.24, 182.74, 147.78, 146.03, 145.96, 134.16, 133.99, 133.74, 133.65, 133.30, 132.63, 131.84, 131.62, 128.35, 128.12, 127.26, 127.21, 126.14, 124.88, 123.72, 116.47.


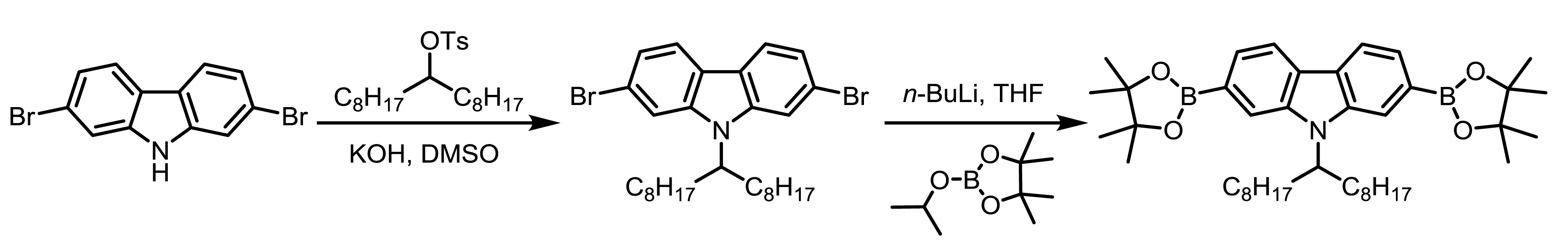


**Scheme S2**. Synthetic pathway of the carbazole monomer.

**2,7-Dibromo-9-(heptadecan-9-yl)carbazole**

2,7-Dibromocarbazole (11.0 g, 34 mmol) and potassium hydroxide (19.0 g, 340 mmol) were dissolved in dry DMSO (90 mL). The reaction mixture was stirred at room temperature for 2 h, and then 18-crown-6 (0.45 g, 1.7 mmol) was added. A solution of 9-heptadecane p-toluenesulfonate (21.0 g, 51 mmol) in 40 mL of dry DMSO was added dropwise. The reaction mixture was stirred at room temperature for 8 h and then heated at 60 °C for 3 h. After cooling, the reaction mixture was poured into 350 mL of water and extracted three times with 100 mL of ethyl acetate, dried over anhydrous sodium sulfate and the solvent was removed under vacuum. The white powder was purified by flash chromatography eluting with petroleum ether and re-crystallized from petroleum ether to afford white solid (17.9 g, 89%). ^1^H NMR (400 MHz, CDCl_3_) δ 7.90 (s, 2H), 7.69 (s, 1H), 7.53 (s, 1H), 7.33 (d, *J* = 6.4 Hz, 2H), 4.41 (tt, *J* = 10.1, 5.1 Hz, 1H), 2.26–2.13 (m, 2H), 1.95–1.84 (m, 2H), 1.29–1.07 (m, 22H), 1.04–0.91 (m, 2H), 0.83 (t, *J* = 7.0 Hz, 6H).

**9-(Heptadecan-9-yl)-2,7-bis(4,4,5,5-tetramethyl-1,3,2-dioxaborane-2-yl)carbazole**

*n*-Butyllithium (15 mL, 2.5M in hexane) was slowly added to a degassed solution of 2,7-Dibromo-9-(heptadecan-9-yl)carbazole (8.4 g，15 mmol) in THF (80 mL) at −78 °C, and the solution was stirred at −78 °C for 1 h. 2-Isopropoxy-4,4,5,5-tetramethyl-1,3,2-dioxaborolane (9 mL，50 mmol) was added to the reaction vessel at −78 °C. The reaction mixture was slowly warmed to room temperature. After stirred overnight at room temperature, the mixture was poured into deionized water (100 mL) and the aqueous layer was extracted with ethyl ether (3 × 60 mL). The organic phases were collected and washed with the deionized water (3 × 40 mL), dried over anhydrous Na_2_SO_4_, filtrated and concentrated under reduced pressure to give the crude product. The residue was purified by column chromatography on silica gel eluting with petroleum ether/ethyl acetate (5:1, v/v), and re-crystalized from ethanol to give pure product as a white crystal (8.5 g, 87%). ^1^H NMR (400 MHz, CDCl_3_) δ 8.12 (s, 2H), 8.02 (s, 1H), 7.88 (s, 1H), 7.66 (d, *J* = 7.6 Hz, 2H), 4.69 (tt, *J* = 10.1, 5.2 Hz, 1H), 2.42–2.25 (m, 2H), 1.93 (m, 2H), 1.39 (s, 24H), 1.17 (m, 22H), 1.04–0.91 (m, 2H), 0.81 (t, *J* = 7.0 Hz, 6H).

**General procedures for the preparation of TADF polymers.**

The polymerization was performed according to general procedures of palladium(II) catalyzed Suzuki polycondensation：Under argon atmosphere, a solution of M1 and M2 (0.5 mmol in total, with the feed molar ratios of 0.5:49.5, 1:49, 2:48 and 5:45), M3 (279 mg, 0.500 mmol) or M4 (329 mg, 0.500 mmol), Pd(OAc)_2_ (3 mg), and PCy_3_ (6 mg) in toluene (10 mL), was stirred and gradually warmed up to 82 °C. Deoxygenated solution of Et_4_NOH (2 mL) in deionized water (2 mL) was added. After stirred at 82 °C for 18 h, phenyl boronic acid (6.0 mg) in THF (1 mL) was added and stirred at 82 °C for 4 h. Bromobenzene (1 mL) was subsequently added and stirred at 82 °C for 4 h. After cooling, the resulting solution was poured into methanol (200 mL), there are red solid to precipitate. After filtered off and dried under vacuo, the red product was dissolved in toluene (20 mL), and then sodium diethyldithiocarbamate trihydrate (0.5 g) and deionized water (8 mL) were added into the mixture. The resulting solution was vigorously stirred at 90 °C for 18 h. After cooling, the resulting mixture was extracted with chloroform, washed with deionized water and dried over anhydrous sodium sulfate, After concentration, the resulting polymer was dissolved in small amount of chloroform, then precipitated in methanol (200 mL) and collected via filtration. The residual filament-like solid was loaded into Soxhlet extractor, and washed with acetone (150 mL) for 16 h. The remaining polymer was dried under vacuo, yields: 77-83%.

PFSOTAQ0.5: M1 (296 mg, 0.495 mmol), M2 (3.2 mg, 0.005 mmol). ^1^H NMR (400 MHz, CDCl_3_) δ 8.14–7.56 ((br, Ar H), 7.50–7.07 ((br, Ar H), 2.77 (s, CH_2_), 2.05 (s, CH_2_), 1.55 (s, CH_2_), 1.41–0.94 (br, CH_2_), 0.93–0.40 (br, CH_2_).

PFSOTAQ1: M1 (293 mg, 0.490 mmol), M2 (6.5 mg, 0.010 mmol). ^1^H NMR (400 MHz, CDCl_3_) δ 8.58 (s, Ar H), 8.37 (s, Ar H), 8.14–7.56 ((br, Ar H), 7.50–7.07 ((br, Ar H), 2.77 (s, CH_2_), 2.05 (s, CH_2_), 1.55 (s, CH_2_), 1.41–0.94 (br, CH_2_), 0.93–0.40 (br, CH_2_).

PFSOTAQ2: M1 (287 mg, 0.480 mmol), M2 (12.9 mg, 0.020 mmol). ^1^H NMR (400 MHz, CDCl_3_) δ 8.58 (s, Ar H), 8.38 (d, Ar H), 8.10–7.55 (br, Ar H), 7.50–7.08 (br, Ar H), 2.77 (s, CH_2_), 2.04 (s, CH_2_), 1.55 (s, CH_2_), 1.44–0.96 (br, CH_2_), 0.93–0.41 (br, CH_2_).

PFSOTAQ5: M1 (269 mg, 0.450 mmol), M2 (32.3 mg, 0.050 mmol). ^1^H NMR (400 MHz, CDCl_3_) δ 8.56 (d, Ar H), 8.38 (dd, Ar H), 8.08–7.99 (br, Ar H), 7.96–7.57 (br, Ar H), 7.22–7.25 (br, Ar H), 2.77 (s, CH_2_), 2.04 (s, 2 CH_2_), 1.53 (s, CH_2_), 1.40–0.95 (br, CH_2_), 0.94–0.49 (br, CH_2_).

PCSOTAQ0.5: M1 (296 mg, 0.495 mmol), M2 (3.2 mg, 0.005 mmol). ^1^H NMR (400 MHz, CDCl_3_) δ 8.59 (s, 1H), 8.43–8.09 (br, Ar H), 7.95–7.69 (br, Ar H), 7.66 (s, Ar H), 7.54 (s, 1H), 7.49–7.32 (br, Ar H), 7.24 (s, 1H), 4.61 (s, CH), 2.80 (s, CH_2_), 2.31 (s, CH_2_), 1.98 (s, CH_2_), 1.64 (s, CH_2_), 1.43–0.94 (br, CH_2_), 0.94–0.72 (br, CH_2_).

PCSOTAQ1: M1 (293 mg, 0.490 mmol), M2 (6.5 mg, 0.010 mmol). ^1^H NMR (400 MHz, CDCl_3_) δ 8.58 (s, Ar H), 8.44–8.32 (br, Ar H), 7.94–7.69 (br, Ar H), 7.66 (s, Ar H), 7.54 (s, Ar H), 7.49–7.31 (br, Ar H), 7.24 (s, Ar H), 4.61 (s, CH), 2.80 (s, CH_2_), 2.31 (s, CH_2_), 1.98 (s, CH_2_), 1.64 (s, CH_2_), 1.43–0.94 (br, CH_2_), 0.94–0.72 (br, CH_2_).

PCSOTAQ2: M1 (287 mg, 0.480 mmol), M2 (12.9 mg, 0.020 mmol). ^1^H NMR (400 MHz, CDCl_3_) δ 8.58 (s, Ar H), 8.44–8.32 (br, Ar H), 8.22 (dd, Ar H), 8.05 (d, Ar H), 7.94–7.68 (br, Ar H), 7.66 (s, Ar H), 7.54 (s, Ar H), 7.49–7.31 (br, Ar H), 7.24 (s, Ar H), 4.61 (s, CH), 2.80 (s, CH_2_), 2.31 (s, CH_2_), 1.98 (s, CH_2_), 1.64 (s, CH_2_), 1.43–0.94 (br, CH_2_), 0.94–0.72 (br, CH_2_).

PCSOTAQ5: M1 (269 mg, 0.450 mmol), M2 (32.3 mg, 0.050 mmol). ^1^H NMR (400 MHz, CDCl_3_) δ 8.51 (s, Ar H), 8.36–8.26 (br, Ar H), 8.25–8.03 (br, Ar H), 7.98 (d, Ar H), 7.89–7.61 (br, Ar H), 7.59 (s, Ar H), 7.47 (s, Ar H), 7.30 (s, Ar H), 7.17 (s, Ar H), 4.54 (s, CH), 2.62 (s, CH_2_), 2.24 (s, CH_2_), 1.91 (s, CH_2_), 1.57 (s, CH_2_), 1.36–0.88 (br, CH_2_), 0.88–0.47 (br, CH_2_).

**
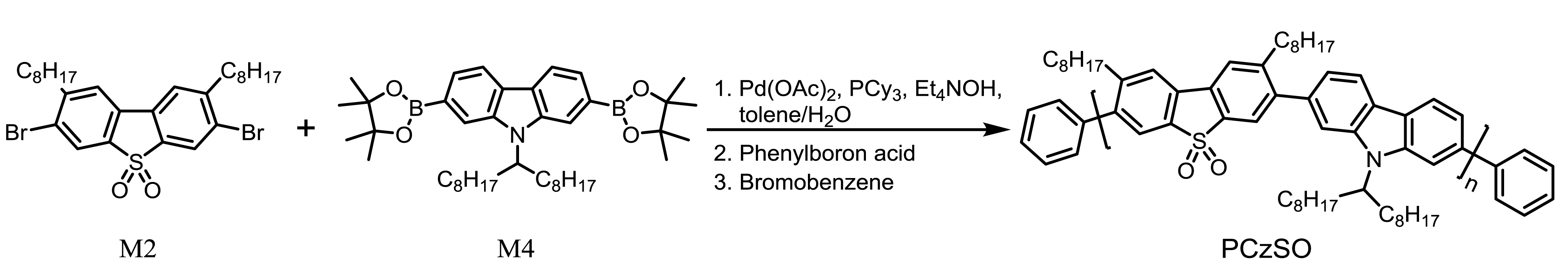
**

**Scheme S3**. Synthetic pathway of the polymer host PCzSO.

PCzSO: M2 (239 mg, 0.4 mmol), M4 (263 mg, 0.4mmol). ^1^H NMR (400 MHz, CDCl_3_) δ 8.23 (d, *J* = 7.1 Hz, Ar H), 8.20 (d, *J* = 7.0 Hz, Ar H), 7.80–7.85 (m, Ar H), 7.53–7.57 (br, Ar H), 7.37–7.42 (br, Ar H), 7.17–7.28 (m, Ar H), 4.61 (s, CH), 2.76–0.84 (m, CH_2_), 2.27–2.34 (m, CH_2_), 1.95–2.02 (m, CH_2_), 1.50–1.56 (m, CH_2_), 1.10–1.30 (m, CH_2_), 0.80–0.92 (m, CH_2_).

**Optical measurements**: The concentration of the solution samples is 10^-5^ M in toluene. Thin film samples were deposited on quartz glass substrates by spin-coating. UV-visible (UV-vis) absorption and Steady State photoluminescence spectra were measured with a Perkin-Elmer Lambda 35 UV-vis spectrometer and a Perkin-Elmer LS 50B spectrofluorometer, respectively. Absolute quantum efficiencies were measured by HAMAMATSU C9920 with an integration sphere. Fluorescence lifetimes were carried out with Edinburgh fluorescence spectrometer (FLSP920) and measured using picosecond pulsed diode laser under the excitation at 375 nm.

**Theoretical Calculation**: All calculations were performed using the *Gaussian* 09 package [3]. The ground-state (S_0_) geometries of two model compounds were first optimized using DFT/B3LYP functional with 6-31G(d) basis set as an initial guess [4]. The calculated charge-transfer (CT) amount (q) was analyzed by *Multiwfn*. The optimal Hartree-Fock percentage (OHF) has an empirical relationship with *q* (OHF = 42*q*) [5]. In view of the calculated OHF value, BMK functional was chosen as the optimal functional [6]. Therefore, the geometries of S_0_ and the lowest excited-state (S_1_) were re-optimized with DFT/BMK and TD-DFT/BMK in toluene solvent, respectively. Related theoretical calculation results are listed in in Table S3 and S4.

**Electrochemical measurements**: Cyclic Voltammetry experiments were performed on an EG&G 283 (Princeton Applied Research). The films of PFSOTAQx and PCzSOTAQx were tested in acetonitrile using ferrocene as an internal reference and n-Bu_4_NPF_6_ as the supporting electrolyte. The HOMO energy levels were calculated according to the equation: *E*_HOMO_ = -[*E*_ox_ - *E*(Fc/Fc^+^) + 4.8] (eV), *E*(Fc/Fc^+^) = 0.34 V.

**Device fabrication and measurement**: The ITO coated substrate was cleaned successively with acetone and ethanol in an ultrasonic bath. After treated with UV-ozone for 20 min, the substrate was coated with a layer of PEDOT:PSS for hole injection and then annealed at 120 °C for 10 min. The emitting layer dissolved in chlorobenzene were then spin-coated onto the PEDOT:PSS layer. After annealed at 50 °C for 10 min, the sample was loaded into a vacuum chamber. The electron transporting layer (SPPO13), the electron injecting layer (Liq) and Al cathode were consecutively evaporated in the vacuum chamber. The EL properties of the devices were measured at room temperature under ambient condition. A PR650 spectra colorimeter (Photo Research) combined with a Keithley 2400/2000 source meter was controlled with the customized software to simultaneously record the current-voltage-luminance characteristics and the EL spectra.

**Table S1**. GPC and TGA results for all TADF conjugated polymers.

| Polymers | *M*_n_ (kDa) | *M*_w_ (kDa) | PDI | T_d_ ( °C) |
| --- | --- | --- | --- | --- |
| PFSOTAQ0.5 | 60.3 | 125.8 | 2.09 | 426 |
| PFSOTAQ1 | 65.8 | 142.6 | 2.17 | 426 |
| PFSOTAQ2 | 54.8 | 111.3 | 2.03 | 432 |
| PFSOTAQ5 | 54.2 | 122.0 | 2.25 | 432 |
| PCzSOTAQ0.5 | 28.8 | 56.4 | 1.96 | 456 |
| PCzSOTAQ1 | 39.1 | 71.2 | 1.82 | 454 |
| PCzSOTAQ2 | 25.4 | 58.0 | 2.28 | 456 |
| PCzSOTAQ5 | 37.2 | 86.9 | 2.34 | 454 |


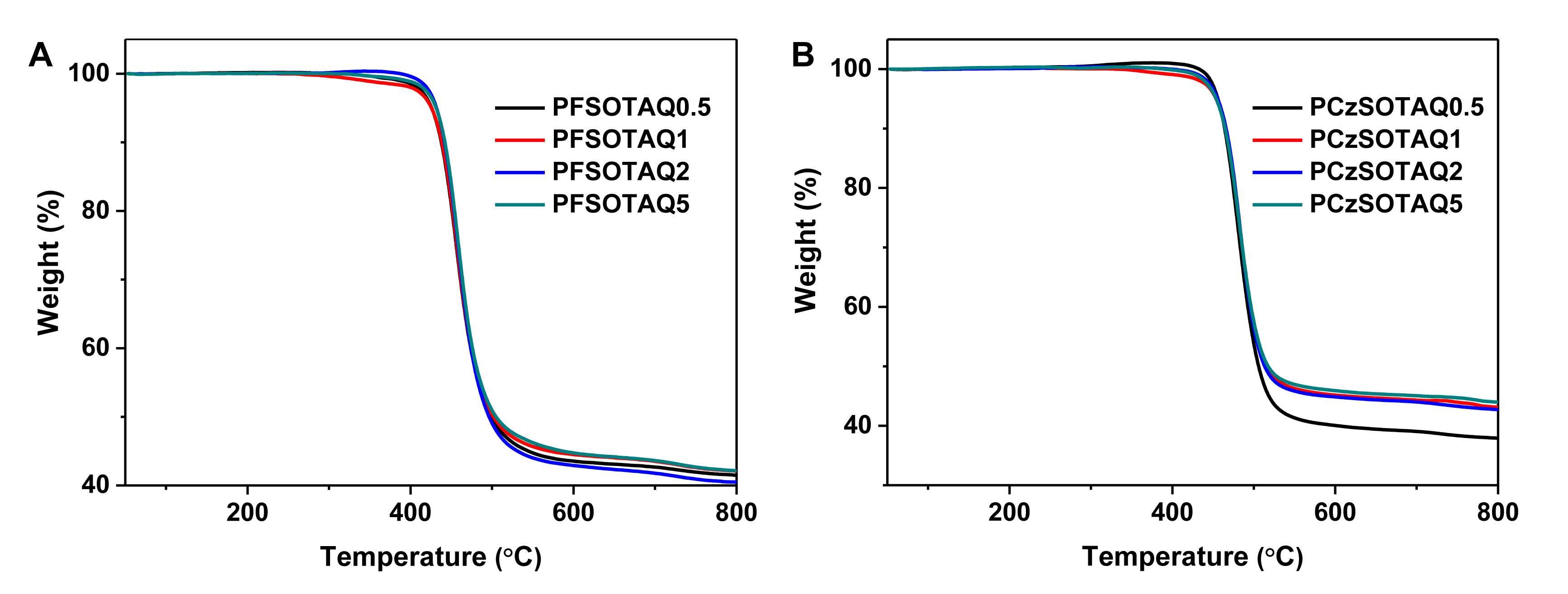


**Figure S1**. TGA curves of PFSOTAQx (A) and PCzSOTAQx (B) at a heating rate of 10 °C min^−1^ under N_2_.


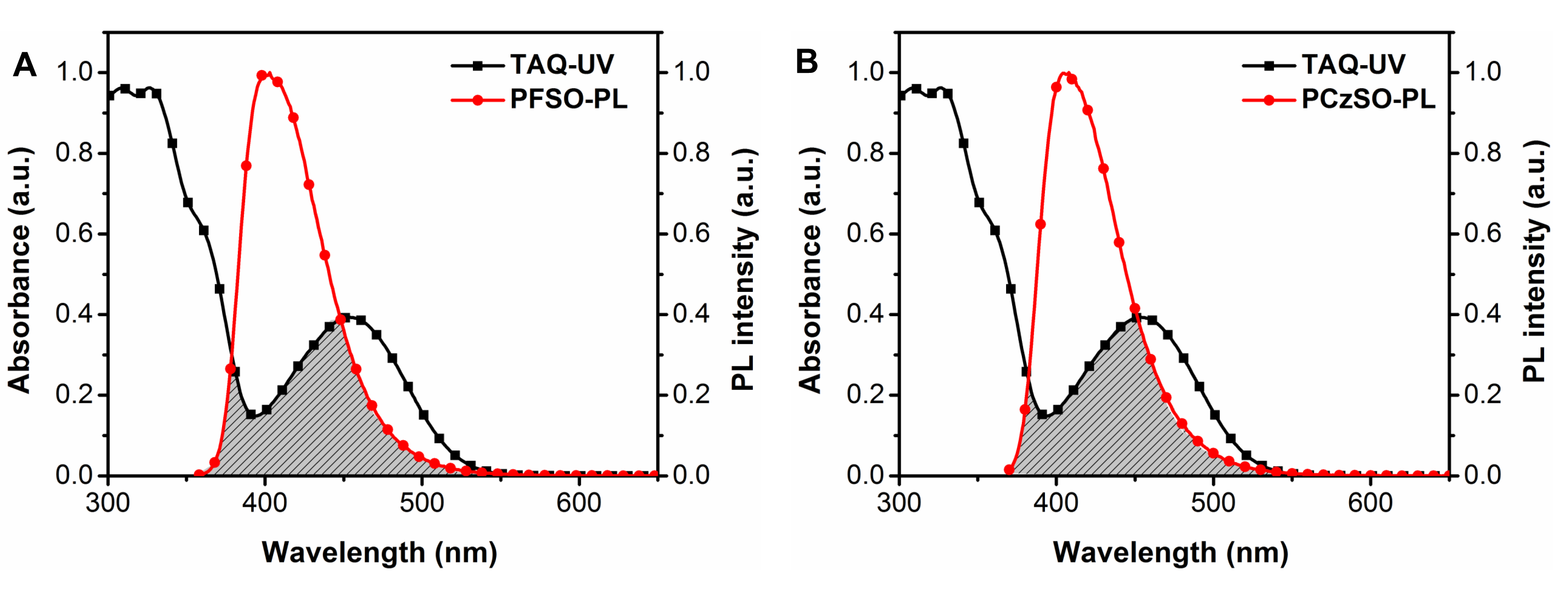


**Figure S2**. UV-vis absorption spectrum of TAQ and PL spectra of PFSO (A) or PCzSO (B) in toluene at 298 K. The shadow part shows their overlap area, which are 28.46 and 28.94, respectively. The PL quantum yields of PFSO and PCzSO are 0.94 and 0.88 in oxygen-free toluene at 298 K, respectively.


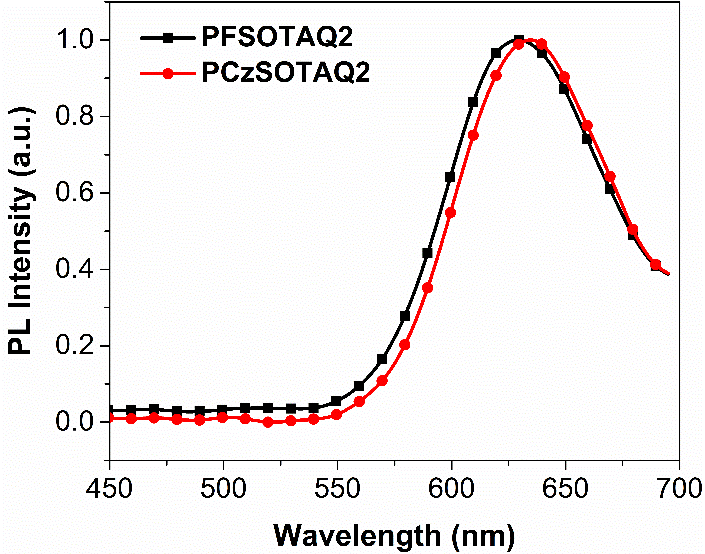


**Figure S3**. Phosphorescent spectra of PFSOTAQ2 and PCzSOTAQ2 in neat film at 77K.

**Table S2** The S_1_ and T_1_ energy levels of PFSOTAQ2 and PCZSOTAQ2 in neat film.

|  | *E*_S_ (eV)^a^ | *E*_T_ (eV)^b^ | Δ*E*_ST_ (eV)^c^ |
| --- | --- | --- | --- |
| PFSOTAQ2 | 2.36 | 2.18 | 0.18 |
| PCzSOTAQ2 | 2.28 | 2.16 | 0.12 |

^a^The singlet (*E*_S_) excited energies estimated from the onset wavelength of the fluorescent spectra in neat film at 298 K; ^b^The triplet (*E*_T_) excited energies estimated from the onset wavelength of the phosphorescent spectra in neat film at 77 K; ^c^∆*E*_ST_ = *E*_S_ - *E*_T_.


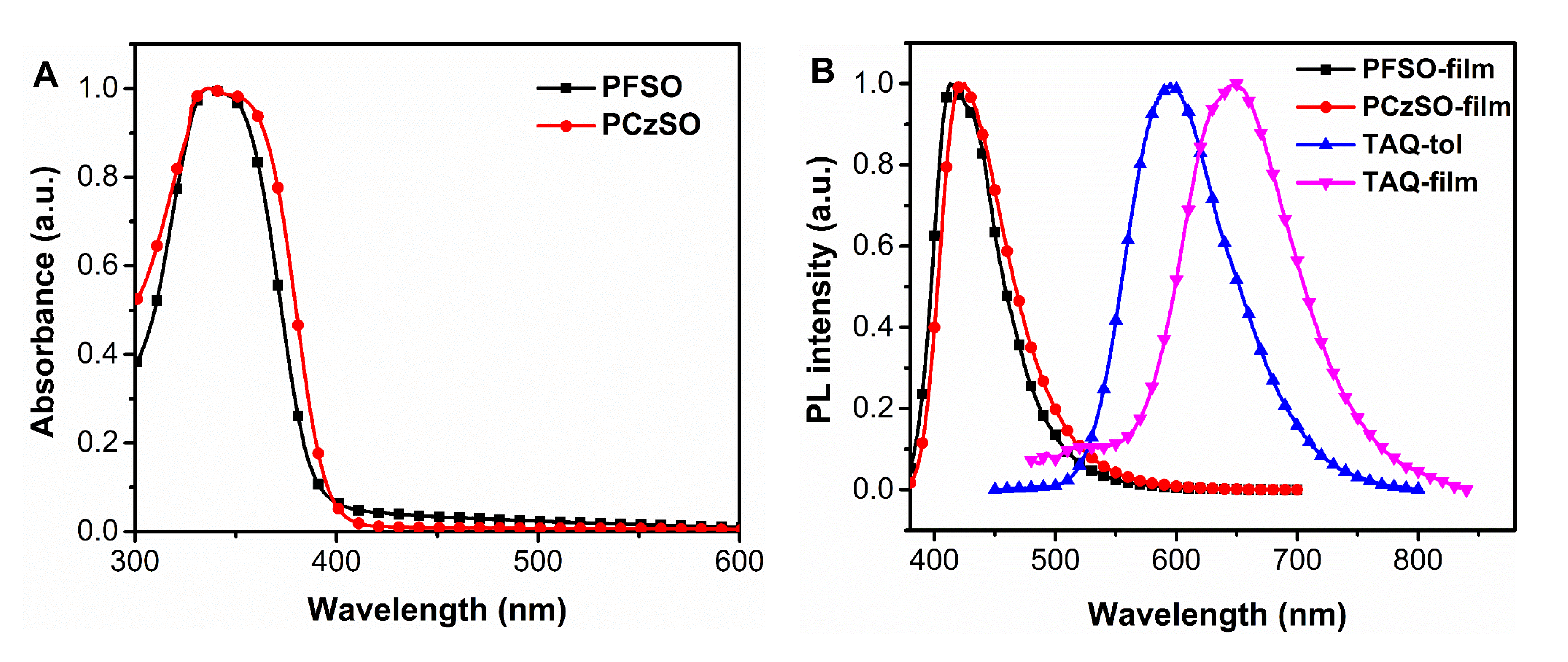


**Figure S4**. (A) UV-vis absorption spectra of PFSO and PCzSO in film; (B) PL spectra of PFSO, PCzSO and TAQ in toluene or neat film at 300 K.


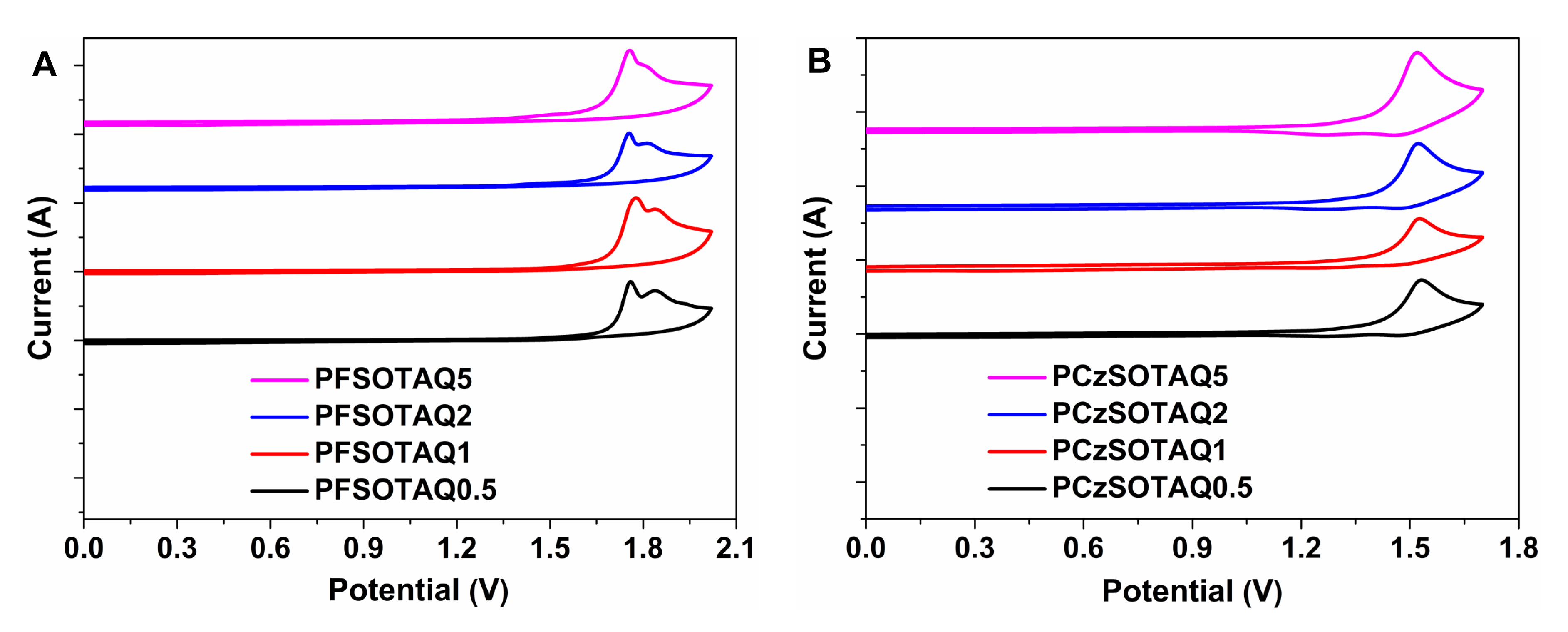


**Figure S5**. Cyclic voltammogram of PFSOTTx (A) and PCzSOTAQx (B).


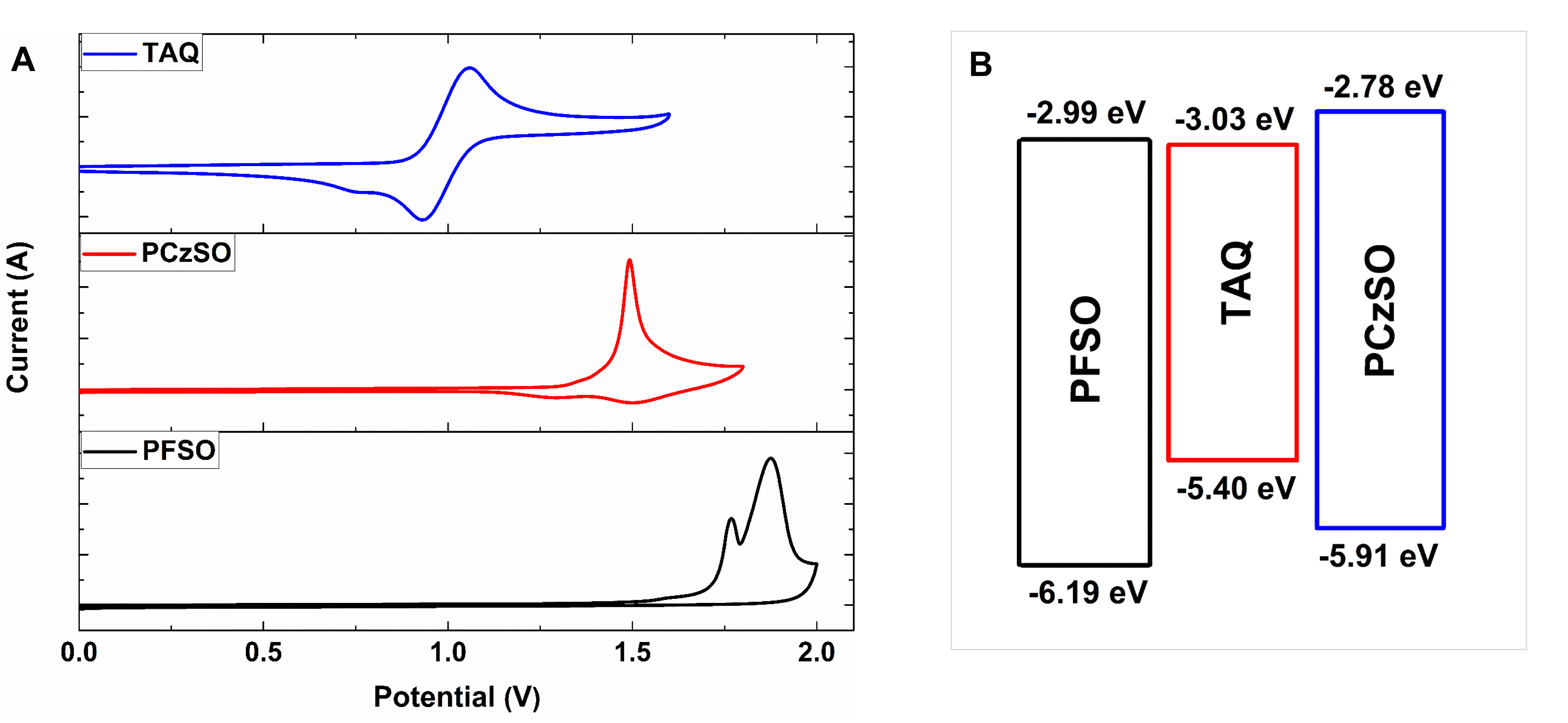


**Figure S6**. (A) Cyclic voltammogram of film state PFSO and PCzSO in acetonitrile, and TAQ in anhydrous dichloromethane; (B) Energy levels of PFSO, PCzSO and TAQ, *E*_HOMO_ = -[*E*_ox_ − 0.34 + 4.8] (eV), *E*_LUMO_ = [*E*_HOMO_ + *E*_g_] (eV), *E*_g_: the optical bandgaps of PFSO, PCzSO in film and TAQ in toluene, *E*_g_ = 1240/λ_onset_.


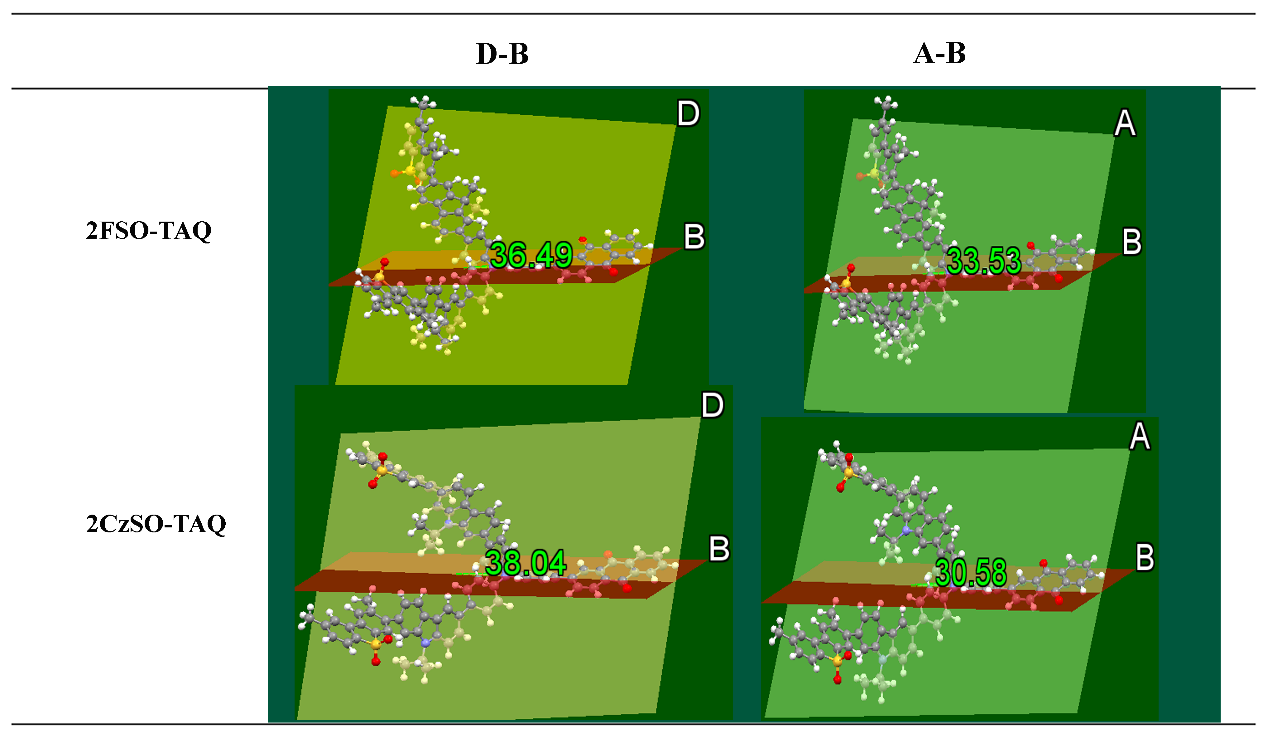


**Figure S7**. The twisting angels between donor (D)/acceptor (A) and phenylene bridge (B) of the polymer models in their S_0_ state in toluene.


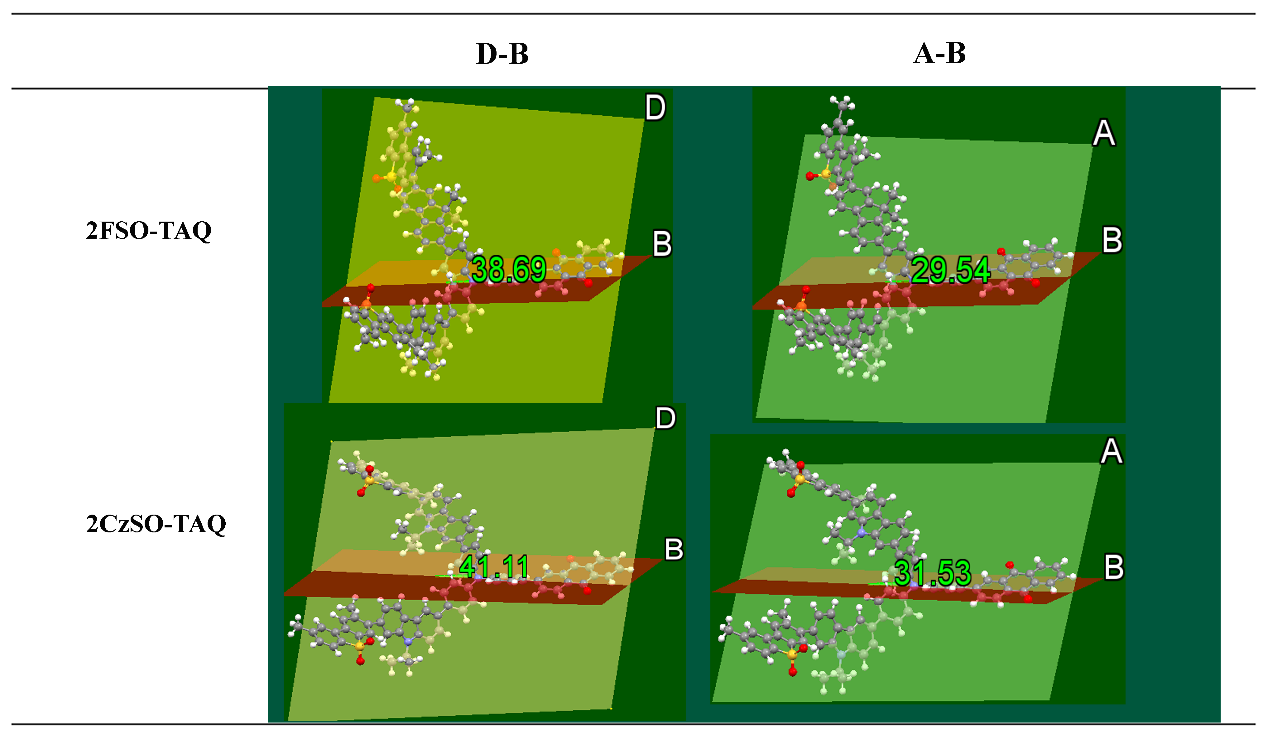


**Figure S8**. The twisting angels between donor (D)/acceptor (A) and phenylene bridge (B) of the polymer models in their S_1_ state in toluene.

**Table S3 Computed CT amount (*q*), optimal HF% (*OHF*), transition energies (*E*_VA_ and *E*_VE_), and zero-zero energies (*E*_0-0_) of the investigated molecules in toluene on S_0_ and S_1_ geometries optimized by the same methods.**

|  | S_0_ geometry in toluene | | |  | S_1_ geometry in toluene | | |  |  |  |  |
| --- | --- | --- | --- | --- | --- | --- | --- | --- | --- | --- | --- |
|  | *q*  (e) | *OHF*  (%) | *E*_VA_(^1^CT)  (eV) |  | *q*  (e) | *OHF*  (%) | *E*_VE_(^1^CT)  (eV) | *E*_0-0_(^1^CT)  (eV) | *E*_0-0_(^3^CT)  (eV) | *E*_0-0_(^3^LE)  (eV) | Δ*E*_ST_/CT  (eV) |
| **2FSO-TAQ** | 0.95572 | 40.14 | 2.73 |  | 0.94264 | 39.59 | 2.35 | 2.54 | 2.34 | 2.43 | 0.20 |
| **2CzSO-TAQ** | 0.88049 | 36.98 | 2.70 |  | 0.95546 | 40.13 | 2.28 | 2.49 | 2.33 | 2.38 | 0.16 |

**Table S4 Computed transition energies (*E*_VA_ and *E*_VE_), oscillator strengths (*f*_VA_ and *f*_VE_) and configuration interaction description of S_1_, T_1_ and T_2_ transitions using TD-B3LYP and TD-BMK, and 6-31G(d) basis set in toluene on the basis of S_0_ and S_1_ geometries optimized by the same methods.**

|  | |  |  | S_0_ geometry in toluene | | | |  | S_1_ geometry in toluene | | | |
| --- | --- | --- | --- | --- | --- | --- | --- | --- | --- | --- | --- | --- |
|  | Functional | |  | *E*_VA_(eV) | CI description | *C_j_%* | *f*_VA_ |  | *E*_VE_(eV) | CI description | *C_j_%* | *f*_VE_ |
| **2FSO-TAQ** | | B3LYP | S_1_ | 1.9147 | HOMO→LUMO | 99.3 | 0.1793 |  | 1.5896 | HOMO→LUMO | 99.5 | 0.1896 |
|  | |  | T_1_ | 1.7904 | HOMO→LUMO | 93.1 | 0.0000 |  | 1.4571 | HOMO→LUMO | 94.3 | 0.0000 |
|  | |  | T_2_ | 2.5479 | HOMO→LUMO+2 | 24.4 | 0.0000 |  |  |  |  |  |
|  | |  |  |  | HOMO→LUMO+4 | 20.1 | 0.0000 |  |  |  |  |  |
|  | |  | T_3_ | 2.5541 | HOMO-12→LUMO | 80.9 | 0.0000 |  |  |  |  |  |
|  | | **BMK** | **S_1_** | **2.7256** | **HOMO→LUMO** | **92.8** | **0.3388** |  | 2.3497 | HOMO→LUMO | 94.4 | 0.3117 |
|  | |  | T_1_ | 2.4313 | HOMO→LUMO | 62.0 | 0.0000 |  | 2.0870 | HOMO→LUMO | 67.9 | 0.0000 |
|  | |  | T_2_ | 2.8052 | HOMO→LUMO | 78.8 | 0.0000 |  |  |  |  |  |
| **2CzSO-TAQ** | | B3LYP | S_1_ | 1.8722 | HOMO→LUMO | 99.1 | 0.1818 |  | 1.4763 | HOMO→LUMO | 99.5 | 0.1714 |
|  | |  | T_1_ | 1.7628 | HOMO→LUMO | 93.2 | 0.0000 |  | 1.3744 | HOMO→LUMO | 95.6 | 0.0000 |
|  | |  | T_2_ | 2.5039 | HOMO-1→LUMO | 58.6 | 0.0000 |  |  |  |  |  |
|  | | **BMK** | **S_1_** | **2.7046** | **HOMO→LUMO** | **92.1** | **0.3627** |  | 2.2751 | HOMO→LUMO | 94.8 | 0.2740 |
|  | |  | T_1_ | 2.4313 | HOMO→LUMO | 62.0 | 0.0000 |  | 2.0712 | HOMO→LUMO | 73.0 | 0.0000 |
|  | |  | T_2_ | 2.8052 | HOMO-10→LUMO | 78.8 | 0.0000 |  |  |  |  |  |

**
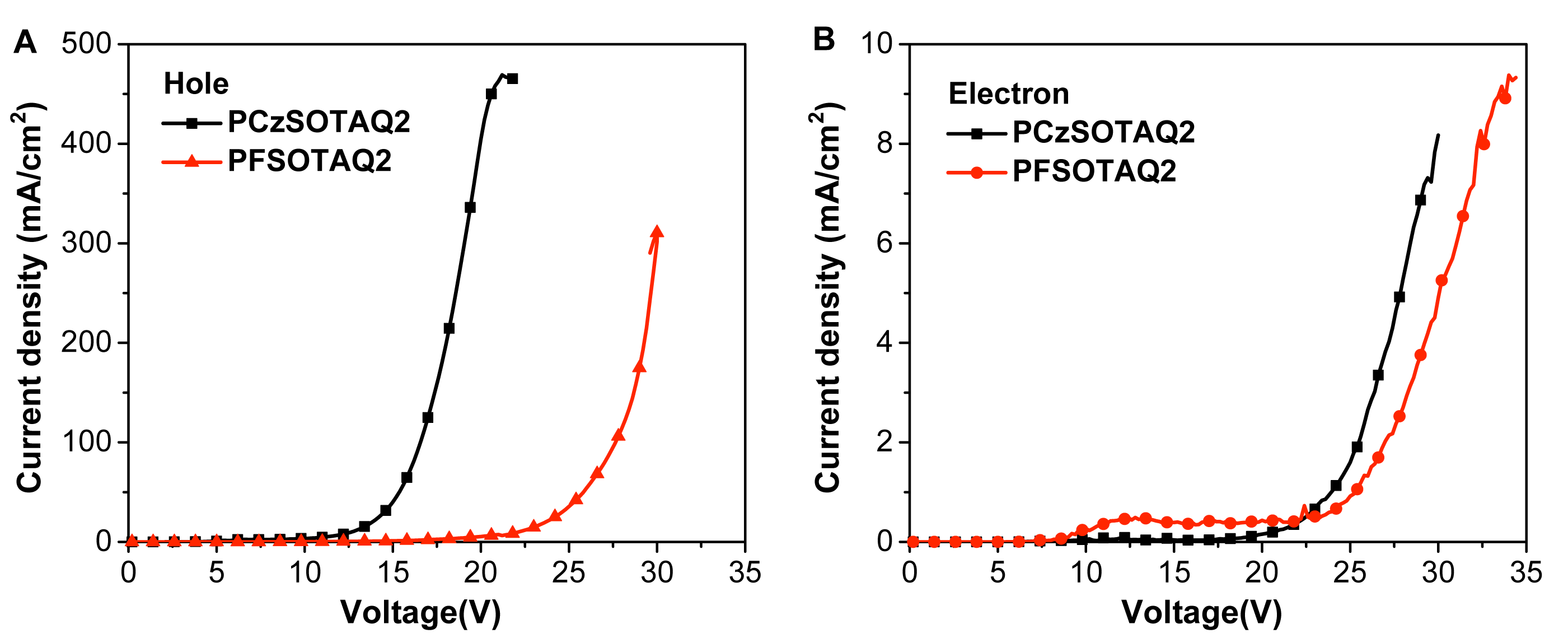
**

**Figure S7**. Current density (*J*)-voltage relationship of hole-only (A) and electron-only (B) devices. Device structures: (A) ITO/PEDOT:PSS(40 nm)/PFSOTAQ2 or PCzSOTAQ2 (40 nm)/MoO3 (10 nm)/Al (100 nm); (B) Al (100 nm)/ PFSOTAQ2 or PCzSOTAQ2 (40 nm)/SPPO13 (60 nm)/LiF (1 nm)/Al (100 nm)

**Table S5** Summary of photophysical parameters for polymers in neat film.

| Polymers | *Φ*_PL_ | *Φ*_F_ | *Φ*_TADF_ | *k*_F_ | *k*_IC_ | *k*_ISC_ | *k*_TADF_ |
| --- | --- | --- | --- | --- | --- | --- | --- |
|  |  |  |  | (×10^7^ S^−1^) | (×10^7^ S^−1^) | (×10^7^ S^−1^) | (×10^4^ S^−1^) |
| PFSOTAQ0.5 | 0.70 | 0.57 | 0.13 | 5.3 | 2.3 | 1.7 | 0.31 |
| PFSOTAQ1 | 0.71 | 0.60 | 0.11 | 5.5 | 2.2 | 1.5 | 0.54 |
| PFSOTAQ2 | 0.58 | 0.49 | 0.09 | 4.8 | 3.5 | 1.5 | 0.63 |
| PFSOTAQ5 | 0.39 | 0.36 | 0.03 | 4.0 | 6.3 | 0.81 | 0.50 |
| PCzSOTAQ0.5 | 0.75 | 0.71 | 0.04 | 6.6 | 2.2 | 0.50 | 1.3 |
| PCzSOTAQ1 | 0.62 | 0.55 | 0.07 | 5.6 | 3.4 | 1.2 | 0.70 |
| PCzSOTAQ2 | 0.53 | 0.46 | 0.08 | 5.2 | 4.6 | 1.5 | 0.67 |
| PCzSOTAQ5 | 0.48 | 0.43 | 0.05 | 4.9 | 5.3 | 1.2 | 0.79 |

The quantum efficiencies for prompt (*Φ*_F_) and delayed (*Φ*_TADF_) components, and the related rate constants including the prompt fluorescence rate (*k*_F_), internal conversion (*k*_IC_), delayed fluorescence rate (*k*_TADF_) and intersystem crossing rate (*k*_ISC_), which were calculated according to the formulas reported by Wang et al.[7]

**
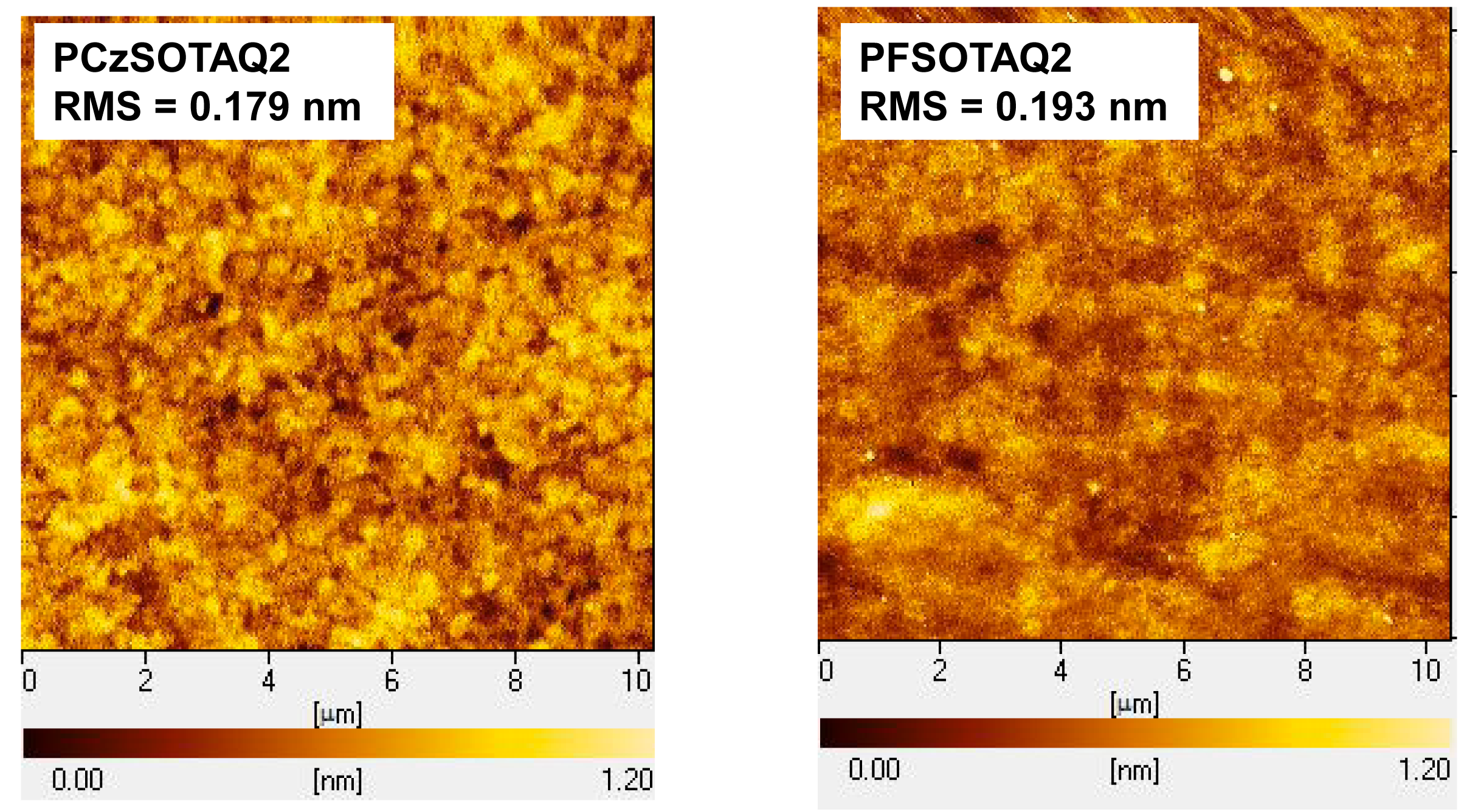
**

**Figure S8**. AFM topographic images of neat films based on PCzSOTAQ2 and PFSOTAQ2.

**References**:

[1] Kamtekar, K. T., Vaughan, H. L., Lyons, B. P., Monkman, A. P., Pandya, S. U., and Bryce, M. R. (2010). Synthesis and spectroscopy of poly(9,9-dioctylfluorene-2,7-diyl-co-2,8- dihexyldibenzothiophene-S,S-dioxide-3,7-diyl)s: solution-processable, deep-blue emitters with a high triplet energy. *Macromolecules* 43, 4481-4488. doi: 10.1021/ma100566p.

[2] Xin, Y., Wen, G.-A., Zeng, W.-J., Zhao, L., Zhu, X.-R., Fan, Q.-L., et al. Huang, W. (2005). Hyperbranched oxadiazole containing polyfluorenes: toward stable blue light PLEDs. *Macromolecules* 38, 6755-6758. doi: 10.1021/ma050833f.

[3] Frisch, M. J., Trucks, G. W., Schlegel, H. B., Scuseria, G. E., Robb, M. A., Cheeseman, J. R. et al. (2009) *Gaussian 09*, Gaussian, Inc., Wallingford, CT.

[4] Reiher, M., Salomon, O., and Hess, B. A. (2001). Reparameterization of hybrid functional based on energy differences of states of different multiplicity. *Theor. Chem. Acc.* 107, 487-455. doi: 10.1007/s00214-001-0300-3.

[5] Zhang, Q., Kuwabara, H., Potscavage, W. J., Jr., Huang, S., Hatae, Y., Shibata, T., and Adachi, C. (2014). Anthraquinone-based intramolecular charge-transfer compounds: computational molecular design, thermally activated delayed fluorescence, and highly efficient red electroluminescence. *J. Am. Chem. Soc.* 136, 18070-18081. doi: 10.1021/ja510144h.

[6] Boese, A. D., and Martin, J. M. (2004). Development of density functionals for thermochemical kinetics. *J. Chem. Phys.* 121, 3405-3416. doi: 10.1063/1.1774975.

[7] Li, C., Duan, R., Liang, B., Han, G., Wang, S., Ye, K., et al. (2017). Deep-red to near-infrared thermally activated delayed fluorescence in organic solid films and electroluminescent devices. *Angew. Chem. Int. Ed.* 56, 11525-11529. doi: 10.1002/anie.201706464.
